# Can the Unified Theory of Acceptance and Use of Technology (UTAUT) Help Explain Subjective Well-Being in Senior Citizens due to Gateball Participation?

**DOI:** 10.3390/ijerph18179015

**Published:** 2021-08-26

**Authors:** Chia-Chien Hsu, Brian Sandford, Chia-Ju Ling, Ching-Torng Lin

**Affiliations:** 1 Department of Tourism, Shih Hsin University, Taipei City 116, Taiwan; hsu127@mail.shu.edu.tw; 2 School of Construction, Pittsburg State University, Pittsburg, KS 66762, USA; bsandford@pittstate.edu; 3 Ph.D. Program in Management, Dayeh University, Changhua 51591, Taiwan; lingchiaju@gmail.com; 4 Department of Information Management, Dayeh University, Changhua 51591, Taiwan

**Keywords:** gateball, leisure participation behavior, senior citizens, subjective well-being, UTAUT

## Abstract

Promoting successful aging strategies through well-reasoned caregiving programs is, and should be, one of the main objectives of many government policies and their implementing agencies. Well-being has been considered an important indicator of successful aging. Leisure is a key life domain and a core ingredient for overall well-being. Yet, within well-being research, few studies have made the connection between leisure participation as accepted behavior and subjective well-being in senior citizens. This study proposed to examine the applicability of the Unified Theory of Acceptance and Use of Technology (UTAUT) in explaining senior citizens’ decision-making processes in terms of leisure participation behavior and the effect of such behavioral engagement on subjective well-being. The respondents were senior citizens in Taiwan who played gateball and were aged 60 years or older. A total of 595 usable responses were obtained and used to answer the research question. The empirical results indicate that performance expectancy, social influence, and facilitating conditions are positively and significantly related to senior citizens’ gateball participation behavior. In addition, gateball participation behavior had a positive and significant effect on respondents’ subjective well-being. The results of this study not only extend the application of UTAUT in terms of participation behavior in leisure activities, but also can provide gateball associations and government entities a theoretical model for developing and promoting gateball programs which serve or involve the elderly, as well as helping older adults to pursue satisfactory levels of wellbeing.

## 1. Introduction

In general, there has been an upward trend in the life spans of the segment of populations that are considered as “aging” commonly encountered in the world. Successfully navigating and experiencing the phenomenon of advancing in age is a goal that older adults in the world are reasonably expected to consider, and perhaps have some meaningful concerns in relation to. It is also an objective of many government policies to address and consider the needs of their aging populations. Social and relational, physical, emotional, and mental well-being have been considered as some of the major and most important indicators of successful aging. As such, the growth in the number of older adults has not only increased public expenditures for this age group, but it also has increased the demand for senior programs and services which provide for the welfare and well-being of this population. The awareness concerning the effects and outcomes of aging are often subtle and can vary by individual. There is the perception that senior citizens require unique and often special attention because of the decline in physical strength, cognitive function, and the narrowing of valuable social networks. However, it has been found that the majority of senior citizens are fit, live independently, and actively engage in a variety of activities [1,2]. How to assist senior citizens to actively engage and adapt in the aging process for the purpose of increasing their quality of life has become a social phenomenon worthy of investigation [3]. Thus, identifying factors that attract and encourage senior citizens to participate in leisure activities which ultimately promote well-being, based upon their own perceptions, is of growing importance.

Leisure activity participation is regarded as a factor that may influence the health of senior citizens [4,5,6]. Research has shown that senior citizens participating in social activities are able to initiate and accrue benefits to their well-being [7]. Using senior citizens in Korea as survey respondents, Ryu and Heo [8] indicated that the relationship between life satisfaction and physical activity participation was positive. The relationship between health perception and outdoor activity participation was also positive. Additionally, in a longitudinal study which investigated the relationship of social and leisure activity profiles and well-being among older adults, Michele et al. [9] found that physical and mental availability played a strong role in the well-being of older adults. Recently, Won et al. [10] used a meta-analysis approach to investigate the psychological impacts of physical activity on the elderly in Korea and indicated that participation in physical activities had an impact on the subjective well-being of senior citizens. Chen and Feeley [1] found that higher degrees of social activity engaged in by older persons may result in higher degrees of individual well-being.

Most of these studies were descriptive or correlational studies. Studies based on theoretical models which examined the leisure participation decision-making process in senior citizens were few. Moreover, studies that foreground a theoretical application emphasizing the participation of senior citizens in a particular leisure activity, and the formation of senior citizens’ subjective well-being, are rare. Previous studies that are of a descriptive or correlational nature have made a contribution in terms of “what” the relationship of leisure activity participation and senior citizens’ subjective well-being is. However, such studies may not have sufficiently addressed the “how” for preventive programs in the health sector for the purpose of helping build effective leisure programs for senior citizens and, subsequently, evaluating the outcomes of those programs in real practice. The advantage of using a theoretical model as a reference is that researchers are able to assess the process of participation perceptual reasoning. There is some ambiguity in terms of how to design and manage data-supported leisure programs to promote leisure participation and, accordingly, to increase subjective well-being as perceived by senior citizens. As such, applying a theoretical model in order to explore the underlying factors resulting in leisure participation behavior and subjective well-being should be helpful in providing a thorough understanding of this particular age group.

The Unified Theory of Acceptance and Use of Technology (UTAUT) was applied to explore the relationship among subjective well-being, leisure participation behavior, and the antecedent variables concerning leisure participation behavior (i.e., performance expectancy, effort expectancy, social influence, and facilitating conditions). Prior research has shown that leisure participation behavior is positively associated with individual well-being [4,5,6]. In the literature, no study has employed leisure participation behavior as a mediating construct between subjective well-being and the constructs, such as performance expectancy and social influence, particularly for senior citizens. This is most likely because senior citizens are often declining in both cognitive and physical functions. The formation of senior citizens’ perception of subjective well-being and their participation in a particular leisure activity, chosen over other alternative activities, is not known. As such, an investigation of the underlying factors which lead to older adults’ leisure activity participation, while examining their subjective well-being, will provide valuable insights into the determinants which drive their leisure participation selections. Furthermore, the results of this study will also provide practical and theoretical implications for health and welfare professionals to consider the motivations for participation in, and thus encourage the development of, leisure programs which encourage leisure participation and hence improve subjective wellbeing for senior citizens.

Arjuna [11] states that the attributes of the elderly include reduced abilities of the five senses, organ disorders, psychological changes, and the emergence of a variety of diseases which reduce the ability of organs to work properly or cause a decline in their function. Thus, Arjuna [11] pointed out that the elderly are not suited for participating in high-intensity sports and should avoid competitions which require physical contact between people. However, there are still many leisure activities well-suited for elderly adults to participate in, such as taking a walk, reading, playing gateball, puzzle solving, conscious resting activities or mindfulness [4], to name a few. Currently, senior citizens in Taiwan have a lot of time available for leisure pursuits. Gateball, which is one of the leisure sports suitable for senior citizens, was invented in Japan in 1947 and introduced to Taiwan in 1982. After more than 30 years of development, through the promotion of gateball associations and teams across the country, the number of gateball players in Taiwan has expanded from only a few dozen in the beginning to more than two hundred thousand as of 2015 [12]. Gateball is a low-intensity sport which is popular among elderly Taiwanese people. Though data on the ages and number of participants are currently unavailable, the majority of gateball participants in Taiwan are over the age of sixty. Gateball is preferred by Taiwanese elderly because this particular leisure activity does not require physical strength or a display of skill. Participation in this activity can fulfill the need for interpersonal or social interactions for elderly people after they retire. For these reasons, this study selected gateball as the leisure activity by which the UTAUT could be used to explore the relationship among subjective well-being and the leisure participation behavior of senior citizens.

## 2. Literature Review and Hypothesis Development

This section addresses how leisure participation behavior is correlated with antecedent variables (i.e., performance expectancy, effort expectancy, social influence, facilitating conditions) and how leisure participation behavior is associated with subjective well-being. Previous studies which relate to the proposed variables are also discussed. 

### 2.1. UTAUT

**Figure 1:**
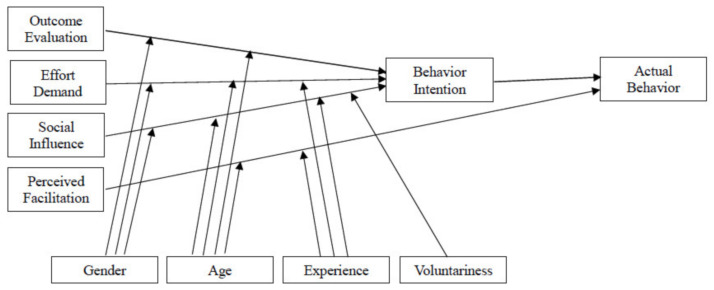
The Unified Technology Acceptance and Use of Technology Theory (Modified from [13]).

Formulated by Venkatesh et al. [13], UTAUT is a modified view of the Technology Acceptance Model, the Theory of Reasoned Action, and the Theory of Planned Behavior. Aiming to explain user intention, and behavior of using a particular information system, this theory presents four antecedent variables related to behavioral intention and, behavioral intention subsequently leads to user behavior (Figure 1). These four antecedent variables are: performance expectancy, effort expectancy, social influence, and facilitating conditions. Performance expectancy, effort expectancy, and social influence directly link to behavioral intention and facilitating conditions are a direct determinant of user behavior. Gender, age, experience, and voluntary use are employed as the moderating variables posited to have an impact on user intention and user behavior. The above moderating variables were not employed in this research effort because the respondents of this study were senior citizens whose ages were 60 or older. The profile of this particular age group might differ from that of previous research studies. 

**Figure 2:**
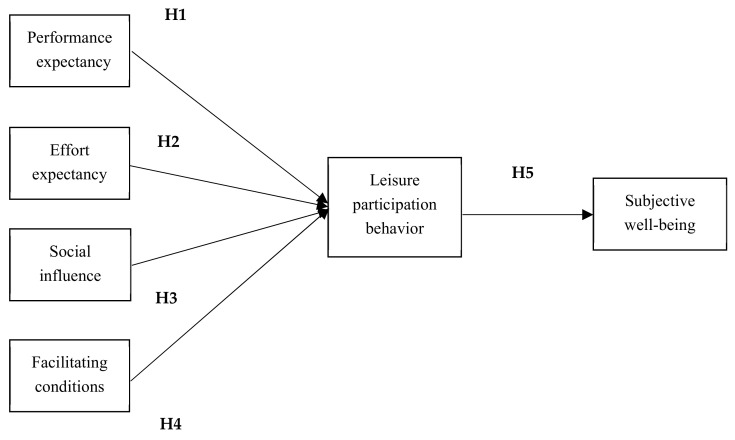
Concept of research model.

The applications of UTAUT mostly focus on various technology adoptions. Eckhardt et al. [14] applied the theory to study the social influence of workplace referents’ on information technology (IT) adoption. Wang and Wang [15] also apply UTAUT to examine user acceptance in terms of mobile internet adoption. Chao [16] applies UTAUT to explore students’ behavioral intention toward using mobile learning. In the field of leisure studies, the application of UTAUT is rare. Lin [17] has proposed a model of leisure activity participation and the results of their study indicate that facilitating conditions, perceived usefulness, and participation intention have significant impact on leisure activity participation. Carlsson et al. [18] employed a developed UTAUT approach to examine a sustained adoption of physical activity programs. The results of this study reveal that a physical activity program should build on activities that younger elderly people feel are meaningful and suitable for their current physical capacity and activity participation history. In this study, the research team also made a modification of the original model. This is because the respondents of this study were gateball players who had participated in this particular leisure activity before. Therefore, the construct of behavioral intention was not included in the hypothetical model since the respondents had already decided to engage in and perform this leisure participation behavior. Figure 2 presents the theoretical framework of the study.

### 2.2. Leisure Participation Behavior

Engaging in leisure activities, also referred to as leisure participation behavior, can help to alleviate individual levels of stress, loneliness, or depression [19,20,21]. Applying the social exchange perspective, persons who are involved in leisure activities are expected to obtain certain kinds of reward(s). Searle [22], and Li et al. [4], documented that leisure participation behavior continues if the rewards of such participation behavior are perceived to be worthwhile, and the opposite is also true; adequate rewards are worthwhile if they are derived from leisure participation. Cheung, et al. [20], studied the relationship between leisure participation behavior and individual well-being. Ragheb and Griffith [23] found that the more leisure participation behavior occurs, the higher the levels of life satisfaction. Other researchers [24,25] found that discontinuing routine leisure activities were likely to have a negative impact on individuals. Those negative impacts are particularly significant for senior citizens. Agahi et al. [26] state that senior citizens may withdraw from the leisure activities they used to be involved in and begin to engage in new ones because their physical abilities and functional status decline. In the context of this study, leisure participation behavior refers to the playing of gateball and its related behaviors. The respondents of the study were senior citizens whose major leisure activity is playing gateball; as gateball is one of the moderate-strength leisure activities suitable for senior citizens. Gateball is a group activity that requires players to communicate with one another and, in turn, is a leisure activity that also includes the social relationship function which can enhance players’ sense of belonging and subjective well-being [27]. 

### 2.3. Performance Expectancy

Performance expectancy is defined as the degree of belief in an individual, when employing a particular system, which will help them perform better [13]. The theoretical base of this construct stems from perceived usefulness (Technology Acceptance Model, TAM), extrinsic motivation (Motivation Model, MM), job-fit (Model of PC Utilization, MPCU), relative advantage (Innovation Diffusion Theory, IDT), and outcome expectation (Social Cognition Theory, SCT) [28]. In the literature, researchers acknowledge that performance expectancy is a term that related and similar to perceived usefulness [13]. In general, performance expectancy is considered to be helpful to a user’s performance in a given task and he/she is more likely to use the performance strategy they have chosen which inversely would increase their performance expectancy. However, from the theoretical viewpoint, this construct may also be influenced by gender and age [13]. In this study’s context, if senior citizens perceive that playing gateball is useful for keeping their mind sharp, maintaining their physical fitness, or providing opportunities for social interaction, they are likely to engage in gateball-playing behavior. Prior research [29,30] has shown that performance expectancy is positively correlated with behavioral intention. This leads to the following hypothesis:

**Hypothesis** **1** **(H1).** *Performance expectancy has a positive influence on senior citizens’ gateball-playing behavior.*

### 2.4. Effort Expectancy

Effort expectancy is defined as the degree of convenience associated with the use of a particular system [13]. The theoretical base of this construct is derived from perceived ease of use (TAM), complexity (MPCU), and ease of use (IDT) [13]. Perceived ease of use is defined as “the degree to which a person believes that using a particular system would be free of effort” [31], p. 320. In fact, effort expectancy is basically identical to perceived ease of use. As used in this study, the construct is approached from a user’s perspective and refers to the degree to which a senior citizen believes that playing gateball requires him/her to exercise little effort. In this context, if senior citizens perceive that playing gateball is not difficult, they are likely to engage in gateball-playing behavior. Prior research [32,33] has also shown that effort expectancy is positively related to behavioral intention. As such, the following hypothesis is formulated:

**Hypothesis** **2** **(H2).** *Effort expectancy has a positive influence on senior citizens’ gateball-playing behavior.*

### 2.5. Social Influence

Based on the subjective norm employed in the Theory of Reasoned Action (TRA) and the Theory of Planned Behavior (TPB), social influence is defined as “the degree to which an individual perceives that others, deemed important to that individual, believe he or she should use the new system” [13], p. 451. The definition presented by Venkatesh et al. [13] is for the exclusive use of information systems. The rationale of social influence lies in the fact that accepted standards of behavior exist in a particular group, community, or culture [4]. The prevailing social influence is generally constituted by salient referents. In real life situations, family members, friends, coworkers, or supervisors are common candidates for the role of salient referent(s). Individuals tend to adopt or perform particular behaviors if they perceive that salient referents prefer certain behaviors [34]. In this study’s context, family members and friends were deemed salient referents. Senior citizens are likely to participate in gateball games or programs if family members and friends are willing to suggest that playing gateball is good for physical fitness and mental health. Prior research [4,35,36] reveals that family members and friends are the most influential persons in shaping one’s viewpoint. This has led to the formulation of the following hypothesis:

**Hypothesis** **3** **(H3).** *Social influence has a positive influence on senior citizens’ gateball-playing behavior.*

### 2.6. Facilitating Conditions

Facilitating conditions refers to “the degree to which an individual believes that an organizational and technical infrastructure exists to support the use of a system” [13], p. 453. The theoretical basis of this construct is derived from perceived behavioral control (Theory of Planned Behavior, TPB), facilitating conditions (MPCU), and compatibility (IDT) [13]. Facilitating conditions may either encourage or hinder the performance of a behavior [37]. If participation in a leisure activity, for instance, is convenient (e.g., in terms of location or low cost) for an individual, he/she is more likely to join in that activity, and vice versa. Facilitation conditions, therefore, may play a pivotal role in whether a person is willing to participate in a leisure activity. In this study’s context, if the gateball field is easy to access or the cost of joining a gateball club is reasonable, the probability of older adults who are willing to play gateball would increase. Conversely, if gateball fields are inconveniently located or joining a club is expensive, only a few older adults would participate in this leisure activity. Prior research [33] has shown that facilitating conditions are positively related to user behavior. This leads to the following hypothesis:

**Hypothesis** **4** **(H4).** *Facilitating conditions have a positive influence on senior citizens’ gateball-playing behavior.*

### 2.7. Subjective Well-Being

The concept of well-being is individually determined by a person’s self-assessment regarding the items related to life concerns [38]. This concept is viewed as a cognitive and judgmental process for the purpose of evaluating a person’s quality of life based on individually chosen criteria [39,40]. Subjective well-being is regarded as a subjective measure of a person’s life. This variable reveals a unique problem due to the fact that the nature of well-being is subject to interpretation. Many persons may address certain life domains (e.g., work, leisure) while other individuals may focus on something vastly different (e.g., individual relationships and/or family life). In fact, the idea of subjective well-being tends to focus on a positive outlook as perceived by the individual. However, researchers [41] state that the measurement of subjective well-being has not received unified acceptance. Global life satisfaction, domain life satisfaction, and positive affect are three different dimensions related to subjective well-being, developed by Xu and Roberts [42]. Ryff and Keyes [43] group subjective well-being into six dimensions. These dimensions include self-acceptance, positive relations with others, autonomy, environmental mastery, purpose of life, and personal growth. In sum, it can be difficult to determine the cutoff point between “too much” and “too little” concerning each individual’s preferences and perceptions related to “well-being” [44].

### 2.8. Leisure Participation Behavior and Subjective Well-Being

Leitner and Leitner [3] indicated that the psychological benefit of leisure participation behavior is one of the key topics addressed in the field of leisure studies. Many researchers believe that the psychological benefit is an important assessment criterion related to leisure activity participation [45] and is associated with the measure of quality of life [46,47,48]. In our context, it was assumed that engaging in gateball-playing behavior is beneficial for senior citizens. Gateball is a game of strategic deployment and it provides exercise, not only for the body but also for the mind [49]. Senior citizens can join this particular activity as a means to maintain their body fitness and to keep their mind active.

Xu and Roberts [42] revealed that perceived well-being has a positive relationship with individual health. Specifically, a positive perception of individual well-being can serve as a promoting agent for one’s health. Employing regular exercises as part of leisure participation behavior, Hassmen et al. [50] pointed out that leisure participation behavior is positively associated with psychological benefits. A meta-analysis conducted by Netz et al. [51] indicated that participation in physical activity is positively linked to perceived well-being. As stated above, prior research has shown that the relationship between leisure participation behavior and subjective well-being is positive and therefore leads to the following hypothesis:

**Hypothesis** **5** **(H5).** *Gateball-playing behavior have a positive influence on senior citizens’ subjective well-being.*

## 3. Method

### 3.1. Measures

A questionnaire was developed for the purpose of examining the hypotheses of the study. A total of 39 statements were developed, based upon theory and an extensive review of the literature. The wordings of those measures were modified for the purpose of being appropriate for this study. The refinement of the instrument was also reviewed by a panel of experts (*n* = 5). Concerning the response categories of this study, a seven point Liker-type scale (1 = strongly disagree, 2 = disagree, 3 = somewhat disagree, 4 = neutral, 5 = somewhat agree, 6 = Agree, 7 = strongly agree) was employed to measure all the constructs of this study. The questionnaire consisted of three sections. Section 1 was intended to assess performance expectancy, effort expectancy, social influence, and facilitating conditions. Section 2 included statements designed to assess predictor constructs (i.e., leisure participation behavior and subjective well-being). Section 3 consisted of statements used to collect demographic information. 

Effort expectancy was measured by seven statements adopted from Davis [31,52], Cardinal [53], Venkatesh and Davis [54], and Venkatesh et al. [13]. Performance expectancy was measured using four statements adopted from Davis [31,52], Cardinal [53], and Venkatesh et al. [13]. Social influence was measured with four statements [4,55,56,57] and facilitating conditions were measured using five statements adopted from Ajzen [58], Taylor and Todd [55], and Li et al. [4]. Based upon the works of Ajzen [59] and Fishbein and Ajzen [60], leisure participation behavior was measured using four statements. The construct of subjective well-being was measured by 15 statements adopted from Ajzen [58], Pouwer et al. [61], Hills and Argyle [62], Chang et al. [2], and Li et al. [4]. 

### 3.2. Data Collection and Sample Profile

Since a complete population list was unavailable, purposeful sampling was used in this study. The respondents were senior citizens in Taiwan who played gateball and were aged 60 years or older. The method of data collection was face-to-face administration. Researchers went to gateball tournaments to ask for senior citizen players to complete the questionnaire. The data collection approach was permitted by the research review committee of one of the investigators’ affiliated institutions. Each respondent was informed that his/her response to the survey questionnaire was totally voluntary and no incentives were provided to the participants. The data collection lasted about three months, from 23 September 2019 to 18 December 2019. As a result, a total of 595 usable responses were used in the data analysis.

SPSS software was employed to analyze the collected data and a total of 595 usable questionnaires were used in the data analysis. The results of the demographic characteristics are listed in Table 1. Of the 595 survey respondents, 63.2% were male (*n* = 313) and 36.8% were female (*n* = 182). Respondents’ age ranged from 60 to 81 years old with the average age being 67.1 years. Middle school (39.4%, *n* = 195) and high school/vocational school (36.8%, *n* = 182) were the major categories regarding their levels of education. Most respondents revealed that they currently lived with their family members (93.7%, *n* = 464) and about 96% of respondents (*n* = 476) revealed that they have played gateball for at least a year. On average, senior citizen gateball players participating in this study responded positively (the mean of each construct is larger than 5.45 out of 7). Table 2 shows the descriptive statistics in terms of the statements of each construct. 

### 3.3. Data Analysis

Statistical Package for the Social Sciences (SPSS) and Analysis of Moment Structures (AMOS) were employed to conduct data analysis. Confirmatory factor analysis (CFA) was first used in order to provide an estimate of a measurement model [63]. Structural equation modeling (SEM) was employed to examine the theoretical model and proposed hypotheses after the adequacy of model was evaluated. In our context, the relationships among subjective well-being, leisure participation behavior, performance expectancy, effort expectancy, social influence, and facilitating conditions were specifically examined. 

The SEM was employed to test the proposed hypotheses. The SEM is used as a multivariable technique which consists of multiple regression analysis and factor analysis. By using the SEM statistic, researchers can assess a series of independent and dependent relationships [64]. AMOS was employed to test the relationships proposed in this study. Confirmatory factor analysis (CFA) was used to screen the possible violations of the linear assumption and to examine if the proposed constructs were valid and reliable. Table 3 presents the results of CFA that include an overview in terms of the average variance extracted (AVE) and correlations among the constructs. The loadings of all statements ranged from 0.63 to 0.96 and exceeded the minimum level of 0.50 suggested by Hair et al. [64]. The results revealed that these statements related to their specified constructs significantly and the unidimensionality of each scale was satisfactory. Composite reliability of each construct ranged from 0.854 to 0.982 and exceeded the value of 0.70 suggested by Bagozzi and Yi [65]. The AVE value was calculated in order to assess the convergent validity of the measures. All but one of the AVE values for each construct exceeded the 0.50 level suggested by Fornell and Larcker [66]. The only AVE value which did not meet the recommended value was subjective well-being with a 0.473 and this was considered acceptable in this case due to its 0.027 proximity to the 0.50 recommendation. The square root values of AVE were used to examine whether the discriminant validity was satisfactory. The diagonal values indicated that the discriminant validity was satisfactory. 

## 4. Results

### Tests of Structural Model

**Figure 3:**
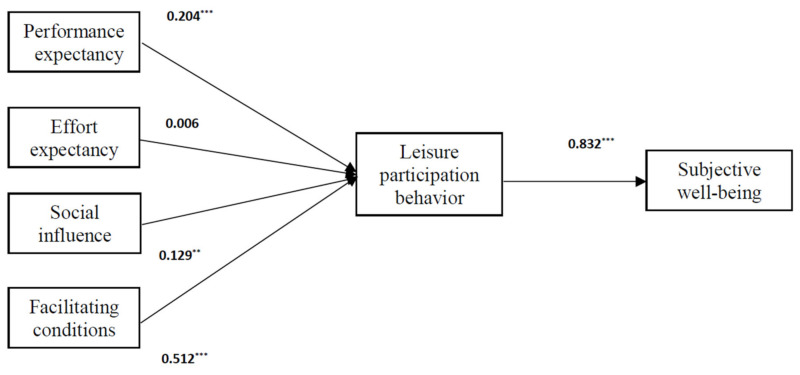
Results of testing hypothetical model (*n* = 595). ** *p* < 0.01, *** *p* < 0.001.

This study was an effort to test the significance of the proposed hypotheses on the basis of the UTAUT. The SEM technique was performed, and the structural model was assessed by χ^2^/df and fit indices. The fit indices consisted of the goodness of fit index (GFI), adjusted goodness of fit index (AGFI), root mean square error of approximation (RMSEA), comparative fit index (CFI), and normed-fit index (NFI) [62]. The model fit was good (χ^2^/df = 2.082, GFI = 0.885, AGFI = 0.870, RMSEA = 0.043, CFI = 0.963, NFI = 0.932). All indicators showed a satisfactory fit between the hypothesized model and the data [64]. Figure 3 shows the results of hypothesis testing. The estimates of the standardized coefficients revealed that the linkages between performance expectancy and leisure participation behavior (β = 0.204, *p* < 0.001), between social influence and leisure participation behavior (β = 0.129, *p* < 0.01), between facilitating conditions and leisure participation behavior (β = 0.512, *p* < 0.001), and between leisure participation behavior and subjective well-being (β = 0.832, *p* < 0.001) were all positive and significant. Therefore, Hypotheses 1, 3, 4, and 5 were supported. However, the linkage between effort expectancy and leisure participation behavior was not significant. Hypothesis 2 was not supported. The findings revealed that performance expectancy, social influence, and facilitating conditions were positively correlated with leisure participation behavior. Based on the estimates of the standardized coefficients (Figure 3), the effect of facilitating conditions on leisure participation behavior was greater than performance expectancy and social influence.

## 5. Discussion and Implications

### 5.1. Discussion

In the field of leisure study, application of the UTAUT model is rare. This study was an effort to examine the applicability of the UTAUT model for explaining senior citizens’ decision making process in terms of leisure participation behavior and the effect of such behavioral engagement on subjective well-being. The results of this study verified that the proposed constructs can be the primary reasons for senior citizens’ participation in gateball activities. Subsequently, their participation behaviors in gateball can lead to senior citizens’ subjective well-being. More specifically, participating in gateball activities may help senior citizens become more positive, as well as leading them toward greater satisfaction with their current quality of life. The findings of this study also indicate that performance expectancy, social influence, and facilitating conditions are all positively and significantly related to senior citizens’ gateball participation behavior. The findings enable researchers to verify the role of antecedent variables and to elaborate the path of the proposed model. Among the antecedent constructs, social influence and facilitating conditions are particularly significant, as addressed by researchers [4,12]. 

The relationship between effort expectancy and leisure participation behavior is insignificant. Thus, effort expectancy, providing the value of ease of use, does not necessarily result in a different level of appeal for gateball activity participation. In order to participate in gateball activities, a senior citizen must first learn the rules and then develop certain skills. Such rules and skills can be learned and developed through a coach or by reading a guidebook. However, being a good gateball player is the result of devoted time, energy, and willingness to interact with others. Senior citizens may evaluate their activities related to gateball participation in these terms, leading to the perception that playing gateball may not be an easy task.

### 5.2. Implications for Practice 

This study provides the following implications contributing to a better understanding of how senior citizens’ decision making processes relate to their leisure participation behavior. Though certain limitations exist, the primary findings of this study consist of practical implications for health and welfare professionals in both the public and private sectors. Principally, health and welfare professionals can use leisure participation behavior (i.e., gateball activity participation) as an option for promoting individual senior citizen well-being. Playing gateball serves as a context for social interaction and provides an opportunity for senior citizens to exercise for the purpose of coping with both mental and physical declines [27]. In fact, gateball is a safe and low-cost activity. It can be performed at a low intensity level and helps senior citizens exercise both their minds and bodies. Such a leisure activity can be employed as a community-based program to further increase the interest of senior citizens to participate in leisure activities.

The gateball program/activity should also be located or available in convenient locations. The findings of this study revealed that facilitating conditions (β = 0.512, *p* < 0.001) had the strongest impact on leisure participation behavior (i.e., gateball activity participation) compared to performance expectancy (β = 0.204, *p* < 0.001) and social influence (β = 0.129, *p* < 0.01). Similar to the results reported by Li et al. [4], these findings imply that senior citizens are likely to participate in gateball activities when they have spare time, feel economically stable, and are able to consider the convenience of location for participating in this particular leisure activity. In addition, the possibility of participating in gateball activities can be improved through social influence [67]. Family members and friends had significant impacts on individual decisions for joining gateball activities. 

### 5.3. Implications for Theory

The original UTAUT model is a parsimonious collection that aims to explain user acceptance and use of information systems [13]. This study was an effort to apply the UTAUT for the purpose of explaining leisure participation behavior and subjective well-being. The construct of behavioral intention was omitted since the respondents of this study were gateball players whose ages were 60 or above and who had already chosen to participate in this leisure activity. These findings reveal that the UTAUT can advance our understanding of leisure activities participation behavior and its effects in senior citizens. In addition, the moderating variables (i.e., gender, age, experience, voluntariness of use) were not included in this study. As Dwivedi et al. [31] stated, “although moderators can be valuable, they may be applicable and become relevant only when there is significant variation in those moderators across individuals within the same context” (p. 729). In this case, the backgrounds of the survey respondents were considered homogeneous and could not reach significant variation. As a result, these moderating variables may not be applicable in this case. Focusing on the direct effects in the UTAUT model can be a better approach towards theory-building [33].

## 6. Conclusions

This paper aims to develop a theoretical explanation concerning the formation of senior citizens’ participation in gateball activities and their subjective well-being. The results revealed that facilitating conditions, performance expectancy, and social influence are positively and significantly associated with leisure participation behavior (i.e., gateball participation behavior). Subsequently, gateball participation behavior has a positive and significant effect on respondents’ subjective well-being. The study applied the UTAUT constructs as the key to developing a reference model for a better understanding of the reasoning process related to leisure participation behavior, particularly for senior citizens. Identical to the result reported by Lin [17], facilitating conditions are the most important construct that impacts leisure participation behavior and these results confirm the UTAUT’s proposition. Since the respondents of the study were active gateball players whose ages were 60 and over, the moderating variables (i.e., gender, age, experience, voluntariness of use) were not applied in this study due to the homogeneity of the respondents. 

This study also leads to some suggestions for further future research. First, purposeful sampling was employed in this study. The results of the study cannot be applicable for activities other than gateball and it is recommended that future research strives to apply the UTAUT model in investigating different leisure activities. Second, due to the homogeneity of the respondents in this study, future researchers could study respondents with more variation in age and experience. Finally, the levels of gateball playing can be different from years of experience and skills learned from practice. As such, many players may pay attention to gateball activities more seriously, whereas some may simply play this particular activity for fun. Currently, this topic has not been fully discussed in the field of leisure study. Future researchers may address this topic more adequately and more specifically.

## Figures and Tables

**Table 1:** Summary of demographic characteristics (*n* = 595).

Variable	Frequency	%
Gender		
Male	313	63.2
Female	182	36.8
Education level		
Middle school	195	39.4
High school/vocational school	182	36.8
College/university	92	18.6
Graduate school	26	5.2
Live with family members		
Yes	464	93.7
No	31	6.3
Playing gateball at least one year		
Yes	476	96.2
No	19	3.8

**Table 2:** Descriptive statistics (*n* = 595).

Variable	Mean	Median	SD	Loading
Performance expectancy				
1. Gateball activity participation helps improve my physical health.	5.45	5	0.75	0.93
2. Gateball activity participation helps me to stay away from loneliness.	5.45	5	0.73	0.94
3. Gateball activity participation provides opportunities for me to interact with other individuals.	5.46	5	0.74	0.94
4. Gateball activity participation helps enjoy my life.	5.47	5	0.72	0.96
5. Gateball activity participation helps improve my satisfaction with life.	5.45	5	0.72	0.93
6. Gateball activity participation helps improve my psychological health.	5.48	5	0.71	0.96
7. Gateball activity participation helps improve my ability to think straight.	5.46	5	0.69	0.93
Effort expectancy				
1. Gateball activity is easy to learn.	5.75	6	0.78	0.85
2. The instruction manual related to gateball activity is easy to understand.	5.7	6	0.75	0.84
3. The rules of gateball activity are easy to understand.	5.74	6	0.76	0.85
4. The skills required for gateball are easy.	5.69	6	0.77	0.83
Social Influence				
1. My family members/friends encourage me to participate in gateball activities.	5.85	6	0.68	0.79
2. To participate in gateball activities, my family members/friends are willing to go with me as companions.	5.69	6	0.73	0.72
3. My family members/friends hope that I can participate in gateball activities frequently.	5.79	6	0.7	0.77
4. My family members/friends believe that gateball is a good leisure activity for me.	5.89	6	0.67	0.8
Facilitating conditions				
1. I have a plenty of spare time to participate in gateball activities.	5.97	6	0.57	0.81
2. I have sufficient economic resources to participate in gateball activities.	5.96	6	0.57	0.78
3. The equipment for gateball activities is convenient for me to carry.	6.06	6	0.5	0.87
4. Gateball fields are convenient located for players.	5.87	6	0.67	0.73
5. A gateball field is conveniently located near my home.	5.87	6	0.66	0.74
Leisure participation behavior				
1. I participate in gateball activities routinely.	6.06	6	0.57	0.82
2. I have participated in gateball activities all along.	6.11	6	0.59	0.91
3. I will continue to participate in gateball activities in the future.	6.13	6	0.58	0.9
4. I will participate in gateball competitions.	6.14	6	0.58	0.89
Subjective well-being				
1. Participating in gateball activities helps me feel a sense of achievement.	5.64	6	0.81	0.66
2. Participating in gateball activities helps me feel a sense of satisfaction..	6.14	6	0.66	0.68
3. Participating in gateball activities helps me feel content with my life.	6.15	6	0.66	0.74
4. Participating in gateball activities helps me think positively.	6.15	6	0.64	0.73
5. Participating in gateball activities makes me happy.	6.15	6	0.66	0.71
6. Participating in gateball activities makes me feel vigorous.	6.12	6	0.65	0.74
7. Participating in gateball activities helps me feel a sense of participation.	6.13	6	0.68	0.73
8. Participating in gateball activities helps enrich my friendship with others.	6.14	6	0.67	0.72
9. Participating in gateball activities helps increase my confidence.	6.13	6	0.65	0.71
10. Participating in gateball activities makes me feel healthy.	6.2	6	0.64	0.65
11. Participating in gateball activities helps enhance my motor skills.	6.09	6	0.72	0.64
12. Participating in gateball activities makes me satisfied with my current life.	6.15	6	0.68	0.7
13. Participating in gateball activities helps me feel the richness of life.	6.13	6	0.7	0.63
14. Participating in gateball activities enables me to improve interpersonal relationships.	6.13	6	0.71	0.63
15. Participating in gateball activities makes me feel rejuvenated.	6.19	6	0.71	0.63

1 = Strongly Disagree, 2 = Disagree, 3 = Somewhat Disagree, 4 = Neutral, 5 = Somewhat Agree, 6 = Agree, 7 = Strongly Agree.

**Table 3:** Measures of correlations, AVE, and composite reliability.

	PE	EE	SI	FC	LPB	SWB	AVE
PE	0.942						0.888
EE	0.76	0.843					0.711
SI	0.65	0.6	0.771				0.594
FC	0.59	0.49	0.76	0.787			0.62
LPB	0.73	0.74	0.65	0.64	0.88		0.774
SWB	0.63	0.68	0.56	0.55	0.66	0.688	0.473
CR	0.982	0.908	0.854	0.891	0.932	0.931	

Note: Diagonals represent the square root of AVEs. PE = performance expectancy; EE = effort expectancy; SI = social influence; FC = facilitating condition; LPB = leisure participation behavior; SWB = subjective well-being; CR = composite reliability.
